# MicroRNA-615-3p decreases apo B expression in human liver cells

**DOI:** 10.1016/j.jlr.2024.100659

**Published:** 2024-09-26

**Authors:** Abulaish Ansari, Pradeep Kumar Yadav, Swati Valmiki, Antoine Laine, Antoine Rimbert, Shahidul Islam, Iman Osman, S. Hani Najafi-Shoushtari, M. Mahmood Hussain

**Affiliations:** 1 Department of Foundations of Medicine, New York University Grossman Long Island School of Medicine, Mineola, NY, USA; 2 Department of Cell and Developmental Biology, Weill Cornell Medicine, New York, NY, USA; 3 Department of Research, Weill Cornell Medicine-Qatar, Qatar Foundation, Education City, Doha, Qatar; 4 Department of Botany, Faculty of Science, University of Allahabad, Prayagraj, India; 5 Nantes Université, CNRS, INSERM, l’institut du thorax, Nantes, France; 6 Department of Medicine, New York University Langone Medical Center, New York, NY, USA; 7 Research Department, New York Harbor Healthcare System, Brooklyn, NY, USA

**Keywords:** apolipoproteins, apoB, lipoproteins, microRNAs, lipids, cardiovascular disease

## Abstract

Plasma lipids are mainly carried in apolipoprotein B (apoB) containing lipoproteins. High levels of these lipoproteins are associated with several metabolic diseases and lowering their plasma levels is associated with reduced incidence of atherosclerotic cardiovascular disease. MicroRNAs (miRs) are small non-coding RNAs that reduce the protein expression of their target mRNAs and are potential therapeutic agents. Here, we identified a novel miR-615-3p that interacts with human 3′-UTR of apoB mRNA, induces post-transcriptional mRNA degradation, and reduces cellular and secreted apoB100 in human hepatoma Huh-7 cells. Reducing cellular miR-615-3p levels by CRISPR-sgRNA increased cellular and secreted apoB100 indicating endogenous miR regulates apoB expression. Overexpression of miR-615-3p along with or without palmitic acid treatment decreased cellular and media apoB and increased cellular triglyceride levels without inducing endoplasmic reticulum stress. These studies have identified miR-615-3p as a negative regulator of apoB expression in human liver-derived cells. It is likely that there are more miRs that regulate apoB-containing lipoprotein assembly and secretion. Discovery of additional miRs may uncover novel mechanisms that control lipoprotein assembly and secretion.

Atherosclerotic cardiovascular disease (ASCVD) is a major cause of global mortality (1). It is commonly associated with high plasma concentrations of cholesterol in low-density lipoproteins (LDL). Lowering plasma LDL reduces the risk of ASCVD. LDL are hydrolyzed products of VLDL primarily synthesized in the liver using an essential structural protein, apolipoprotein B100 (apoB100), and a required chaperone microsomal triglyceride transfer protein (MTP) (2, 3). Several successful interventions have targeted apoB100 and MTP to reduce plasma LDL levels. For example, apoB100 levels have been lowered using antisense oligonucleotides and antisense approaches (4, 5, 6, 7, 8, 9). Similarly, inhibition of MTP’s lipid transfer activity reduces plasma apoB and lipid levels (9, 10). However, these reductions are associated with increases in tissue lipids (8, 9, 11). Therefore, identifying novel mechanisms that regulate these proteins may guide us in novel ways to modulate their expression.

ApoB100 is generally believed to be constitutively transcribed and translated. However, its secretion is regulated by intracellular co-translational and post-translational degradation of newly synthesized protein (2, 12). Nevertheless, there is some emerging evidence to suggest that apoB synthesis and secretion can be modulated at transcriptional and post-transcriptional levels. For example, Vigilin, Tial1, and Hur may modulate apoB mRNA maturation and affect its synthesis and secretion (13). We have previously identified miR-584p as a post-transcriptional regulator of apoB synthesis and secretion (14).

MicroRNAs have emerged as modest, and yet significant, biological modulators of protein function (15, 16). These small non-coding RNAs are present both in plant and animal kingdoms and are derived from endogenous genes. They act as post-transcriptional inhibitors of protein expression. The primary transcripts of the miR genes are processed in the nucleus and exported out as pre-mirs (precursor-mirs) (15, 17, 18, 19, 20). These pre-mirs are further cleaved in the cytoplasm to produce mature miRs (16, 21). MiRs associate with Argonaut (Ago) family of proteins to form an RNA-inducible silencing complex (RISC) that targets specific mRNAs (16). The specific targeting of mRNAs involves the interaction of the 5′-end seed sequence of the miRs (2–7 nucleotide relative to 5′-end) with the 3′-untranslated region (UTR) of mRNAs. The base pairing of miRs with mRNAs in the RISC complex leads to inhibition of mRNA translation and/or increased mRNA degradation (16, 21). These post-transcriptional regulatory mechanisms reduce protein synthesis and, in general, have modest inhibitory effects and therefore simultaneously slow down several metabolic pathways. This is because miRs can simultaneously interact with several different mRNAs that have complementary sequences in their 3′-UTR. Therefore, miRs have a unique ability to interact with several target mRNAs and regulate multiple pathways simultaneously.

Few microRNAs that regulate apoB-containing lipoproteins have been identified (21, 22, 23, 24). We have previously described miR-548p and miR-30c that regulate apoB and MTP, respectively (14, 25). We showed that miR-548p decreases apoB expression by interacting with the 3′-UTR of apoB mRNA (14). Recently, we screened a miRNA library to identify microRNAs that regulate apoB and apoA1 expression and identified miR-541-3p as a potential regulator of plasma LDL and HDL (26). In this study, we continued our search for miRs that regulate apoB and identified miR-615-3p as a novel regulator of apoB expression in human hepatoma Huh-7 cells.

## Materials and Methods

### Cell culture

Human hepatoma Huh-7 cells were cultured in Dulbecco’s modified Eagle’s medium (DMEM) containing 10% fetal bovine serum (FBS), and 1% L-glutamine in T75 flasks (Corning; # 430641U) at 37°C in humidified 5% CO_2_ incubators (27, 28).

### Transfection of hepatoma cells

Huh-7 cells (2.5 × 10^5^) were reverse transfected in 6-well plates using EndoFectin™ Max transfection reagent (GeneCopoeia; #EF013) with indicated doses of miRIDIAN miRNA mimics (miR-615-3p # 4464066, assay id MC11731, miR-615-5p # 4464066, assay id MC12460, Thermo Fisher Scientific), scramble control mimic (20 nM, Thermo Fisher Scientific, #4464058). After 16 h of transfection, the media was replaced with fresh DMEM containing 10% FBS and 1% L-glutamine. For long-term cultures, media was changed every 24 h. A mixture of sgRNAs against *MIR615* gene (synthego, seq 1 CCGGAGGAUUCCAGCGACUC, seq 2 AGGAUUCCAGCGACUCGGGA, seq 3 UCCAGCGACUCGGGAGGGGC) were complexed with Cas9 (Synthego SpCas9 2NLS Nuclease) by mixing 3:1 ratio of sgRNA-miR-615 (3 μmol) and Cas9 (1 μmol) in 5 μl of PBS. This ribonuclear protein (RNP) complex was incubated at room temperature for 20 min. Huh-7 cells (∼80–90% confluent) were dislodged from plates by trypsin treatment, centrifuged at 1,260 rpm or 300 g for 5 min, and cell pellets were suspended in buffer R (Neon transfection system #MPK1096, Thermo Fisher Scientific). Next, 5 μl of RNP complexes were mixed with 10 μl of Huh-7 cells (250,000 cells), aspirated into gold-plated tips (#MPK 1096, Thermo Fisher Scientific), and electroporated at 1,250 V for 10 milliseconds in Neon transfection system (#MPK5000, Thermo Fisher Scientific) (28). The electroporated Huh-7 cells were plated in 6 well plates containing pre-warmed DMEM.

### ApoA1 and apoB protein quantifications in Huh-7 cells and media by ELISA

After 16 h of transfections, the media was replaced with 1 ml of fresh DMEM containing 10% FBS. Media and cells were collected after 48 h for apolipoprotein quantification. Cells were lysed with 0.1 N NaOH and used to quantify total protein by the BCA method (Thermo Fisher Scientific, # 23227**)**. The amounts of the apoA1 and apoB proteins in the media were normalized with total cell protein content. Transfected Huh-7 cells were lysed with 1 × RIPA buffer (Thermo Fisher Scientific, # 89901) containing protease inhibitor mixture (Sigma-Aldrich, #P2714, 1:100 dilution) to measure cellular apoB protein levels by ELISA (27). Cell lysates were centrifuged and the supernatants were added to ELISA plates (Costar, Corning # 3366) to measure apoA1 (R&D Systems® ELISA Kits; # DY3664) and apoB by sandwich ELISA using 1D1 as capture antibodies as described before (27).

### Quantification of mRNAs by quantitative RT-PCR

After 16 h of transfections, the media was replaced with 1 ml of fresh DMEM containing 10% FBS. Media and cells were collected after 24 h for analysis. After washing Huh-7 cells with PBS, 1 ml of TRIzol was added, mixed vigorously for 30 s, and left at room temperature for 10 min. The contents were transferred to microcentrifuge tubes, chloroform (200 μl) was added, mixed by inverting 3–5 times and centrifuged at 12,000 rpm for 15 min at 4°C. The top aqueous phase was transferred to a fresh tube containing equal parts of chilled isopropanol. After 5 min at room temperature, samples were centrifuged (12,000 rpm, 15 min, 4°C), the supernatants were discarded and the pellets were re-suspended in 1 ml of 70% ethanol. Again, samples were centrifuged, supernatants were discarded and the pellets were kept at 37°C for 5–10 min to dry (avoided over drying). Pellets were dissolved in 50 μl of RNase-free water and concentrations were determined by measuring absorbance at 260/280 nm. cDNA was synthesized using 1 μg of RNA and Applied Biosystems™ High-Capacity cDNA Reverse Transcription Kit (Thermo Fisher Scientific; # 4368813). For quantitative RT-PCR, cDNA was diluted 10 times, PowerTrack SYBR Green Master Mix (Thermo Fisher Scientific; # A46109) was used. The Ct values for each mRNA were then normalized to 18S (26, 29).

### Quantification of microRNAs in cells and human plasma by Taqman

Total RNA including miRs was isolated from 250 μl of human plasma using miRNeasy Serum/Plasma Advanced Kit (Qiagen, # 217204), and total RNA was isolated from Huh-7 cells by TRIzol method. RNA (100 ng) and miR-615-3p, RNU44 (Thermo Fisher Scientific, # 4427975, assay id 001094) and U6 (Thermo Fisher Scientific, # 4427975, assay Id 001973) specific primers (Thermo Fisher Scientific, # 4464066, Assay Id MC11731) were utilized to synthesize cDNA for miR quantification using the TaqMan MicroRNA Reverse Transcription kit (Applied Biosystems, 4366597). cDNA obtained from human plasma and Huh-7 cells were used for qRT-PCR to detect miR-615-3p level using TaqMan™ Universal PCR Master Mix (Thermo Fisher Scientific, # 4304437). After normalization with RNU44 (for plasma) or U6 (for Huh-7 cells), the levels of the miR-615-3p were quantified using the ΔΔCt method (30) and are shown as arbitrary units.

### Immunoblotting

Cold 1× RIPA buffer (200 μl) containing protease inhibitor mixture was added to each well of Huh-7 cells cultured in 6-well plates, kept on ice, mixed, transferred to a different tube, vortexed, and subjected to sonication (Branson Digital Sonifier SFX 150, pulse 5 s on and 5 s off, three times) on ice. After complete lysis, cell lysates were centrifuged at 10,000 rpm for 10 min at 4°C. For apoB and β-actin, total proteins (25 μg) were resolved on 6% and 10% acrylamide gels, respectively, transferred to nitrocellulose blotting membranes using the semi-dry transfer method, and then blocked with 5% dry milk powder in Tris-buffered saline (TBS) for one hour. Membranes were washed thrice with TBS that contained 0.1% Tween® 20 detergent (TBST) and then incubated with 1:1000 diluted primary anti-goat polyclonal antibodies against human apoB and anti-rabbit antibody against human/mouse β-actin (Cell signaling, # 4967L) overnight at 4°C. The next day, membranes were washed thrice with TBST and then incubated with alkaline phosphatase-conjugated secondary antibody (1:10,000 dilution) for 2 h at room temperature. After that membranes were washed, the substrate was added and bands were visualized using BioRad ChemiDoc.

### Luciferase assay

Plasmids (GLuc/SEAP dual-reporter vector system) that express wild-type *Gaussia* luciferase gene that contains control 3′-UTR (GeneCopoeia, #CmiT000001-MT05) and *Gaussia* luciferase with the apoB 3′-UTR (GeneCopoeia, #HmiT009218-MT05) were purchased. We performed site-directed mutagenesis of apoB 3′-UTR to disrupt interactions with seed complementary sequences as shown in Fig. 5A using forward TGGCACCAGGatgCGGAAGGTCTC and reverse GCTTTGGTGCAGGTCCAG primers. First, 5 μg of these plasmids were forward transfected into Huh-7 cells. Cells were trypsinized after 24 h of transfection, evenly distributed into wells, and then reverse transfected with miR-615-3p, or electroporated with gRNA-miR-615-3p/Cas9 complexes. After 8 h of reverse transfection or electroporation media was changed, and luciferase activity was assessed in the media obtained after 24 h using a kit. SEAP (secreted alkaline phosphatase) activity was used to normalize luciferase activities.

### Ago2 precipitation

Huh-7 cells transfected with miR-615-3p or gRNA-miR-615-3p were collected and lysed in buffer K (1 mM Tris, 1 mM EGTA, 1 mM MgCl_2_, pH 7.6) by sonication (pulse 5 s on and 5 s off, three times). Cell lysates were centrifuged (10,000 rpm, 10 min, 4°C) and supernatants were precleared by incubating them with IgG for 1 h. Subsequently, samples were mixed with protein A/G plus agarose beads for 1 h at 4°C. This mixture was centrifuged at 800 rpm for 1 min, and the collected supernatant was incubated with 1:100 dilution of human Ago2 antibody overnight at 4°C. The next day, mixtures were incubated with protein A/G agarose beads for 1 h, and centrifuged at 800 rpm for 1 min. Pellets containing agarose beads were processed for RNA isolation using the TRIzol method.

### Preparation of 0.5 mM palmitic acid (PA) and BSA complexes at 5:1 M ratio

PA (41.8 mg) was dissolved in 1 ml of ethanol to make 150 mM stock solution by gentle periodic vortex and heating at 65°C. Separately, we prepared 10% (v/v) fatty acid-free BSA in Milli-Q water and filtered the 10% BSA solution using a 0.2 μm Millipore Filter Unit. Under a laminar flow hood, we aliquoted 67 μl of 10% BSA in a 1.5 ml Eppendorf tube and kept it in 37°C water bath for 5 min. Subsequently, we added 3.3 μl of the 150 mM PA stock solution into 67 μl of 10% BSA and incubated this complex at 37°C water bath for 5 min or until solution visually became transparent. Then 930 μl of culture medium warmed to 37 °C was added.

### Treatment of Huh-7 cells with PA

Huh-7 cells were first transfected with 25 nM of miR-615-3p. After 40 h (for RNA isolation) or 62 h (for protein expression and lipid isolation) of transfection, cells were treated with 0.5 mM of PA during the final 8 h. Media were collected and cells were washed with cold PBS and the lysed for RNA isolation and protein expression.

### Oil Red O staining

Huh-7 cells were stained with Oil red O using a kit (Abcam, ab287838 – Lipid Staining Kit). In brief, Huh-7 cells were transfected with miR-615-3p (25 nM). After 70 h, media was removed from the cells and gently washed 2X with Wash Buffer II/PBS. Next, we added 10% Formalin to each well and incubated for 30 min to 1 h to fix the cells. After removing formalin, cells were gently washed 2X with dH_2_O for 5 min. We then added isopropanol (60%) to each well and incubated for 5 min. After removing isopropanol, we added Oil Red O Working Solution to cover the cells completely and kept them on rotator. After 10–20 min, we removed Oil Red O solution, washed cells 2-5X with dH_2_O, added Hematoxylin, and incubated for 1 min, removed Hematoxylin, and washed with dH_2_O 2-5X. Lipid droplets appear red, and nuclei appear blue. After staining with Hematoxylin and washing with dH_2_O, cells were washed 3X with 60% isopropanol each time for 5 min, with gentle rocking. Next, we extracted the Oil Red O stain by adding 500 μl of 100% isopropanol and incubating for 5 min, with gentle shaking. We used 200 μl to read absorbance at 492 nm. For background subtractions, we used 100% isopropanol.

### Lipid measurements in cells

Huh-7 cells were transfected with miR-615-3p and then treated with palmitic acid (PA) as described before. Cells were washed and incubated overnight with 1 ml of isopropanol at 4°C. Isopropanol was collected, and cells were lysed with 1 ml of 0.1 N NaOH for total protein estimation. Collected isopropanol was used to measure total plasma cholesterol and triglyceride concentrations using kits (Pointe Scientific, Catalog 23-666-411, 23-666-200).

### Statistics

Experiments were performed in biological triplicates and measurements were also done in technical triplicates. Experiments were usually performed three times and data from one representative experiment are presented. Statistical analysis was performed using an unpaired Student *t* test. For comparison between two groups were performed using two-way ANOVA. In the figures, significant differences are represented as ∗*P* < 0.05; ∗∗*P* < 0.01; ∗∗∗*P* < 0.001; ∗∗∗∗*P* < 0.0001.

## Results

### Identification of miR-615-3p as a regulator of apoB

**Fig. 1:**
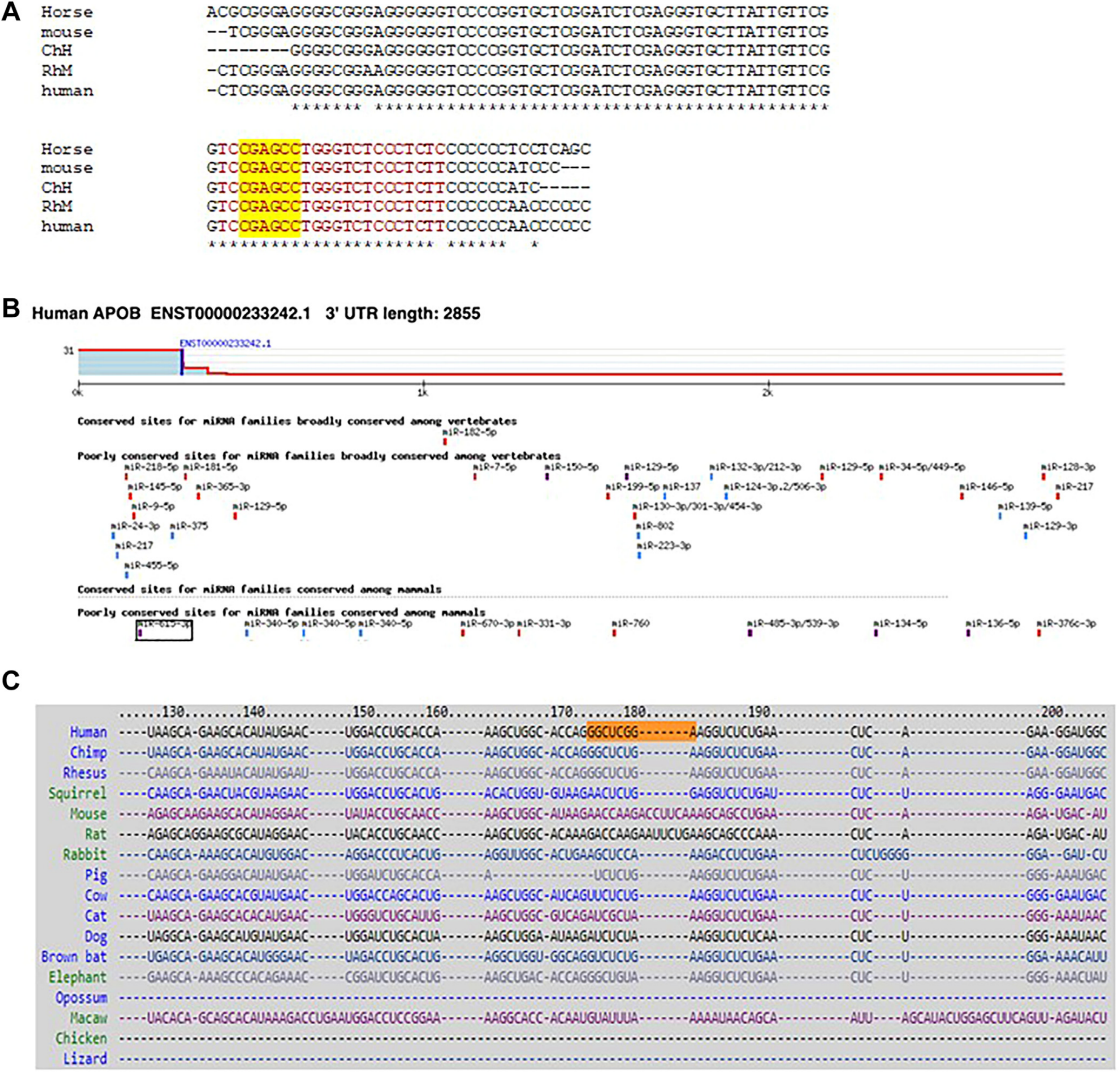
MiR-615-3p is conserved in mammals and can potentially interact with 3′-UTR of human apoB mRNA. A: MiR-615-3p sequences from different animals were aligned. MiR-615-3p is highly conserved (red). The seed sequence has been highlighted in yellow. B: target Scan was queried for the ability of miR-615-3p to recognize human apoB mRNA. This analysis recognized one potential miR-615-3p binding site at 3′-UTR in apoB mRNA that is poorly conserved in mammals. Different colors associated with different microRNAs represent exact matches between the seed sequence of individual miRs with the complementary 3′-UTR in apoB mRNA. Purple rectangles represent an exact match to positions 2–8 of the mature miR followed by an A (8mer). Red rectangle represents an exact match to positions 2–8 of the mature miR (7mer-m8). The blue rectangles represent an exact match of positions 2-7 of the mature miR seed sequence followed by an A (7mer-A1). C: further alignments revealed that only human apoB mRNA 3′-UTR contains a complementary sequence to the seed sequence of miR-615-3p.

Recently we screened a library of human miRs to find potential miRs that could downregulate apoB secretion. In this screen, we found that miR-615-3p reduces apoB secretion by 50% in human hepatoma Huh-7 cells (26). To confirm the possibility of apoB regulation by miR-615-3p and to elucidate mechanisms, we performed in silico analyses. MiR-615-3p is highly conserved in several species with identical seed sequences (Fig. 1A). TargetScan analysis, to query whether apoB mRNA is a target of miR-615-3p, revealed that 3′-UTR of human apoB mRNA contains a miR-615-3p seed complementary sequence that is poorly conserved in mammals (Fig. 1B). Further analysis revealed that miR-615-3p only targets human apoB mRNA and does not recognize apoB mRNA in other mammals and rodents such as chimpanzee, monkey, squirrel, mouse, rat and rabbit (Fig. 1C). Similar analysis using MicroRNA Target Prediction Database (miRDB) also suggested that only human apo B mRNA is a potential target of miR-615-3p (not shown). These studies indicate that the miR-615-3p recognition site in human apoB mRNA might have recently evolved.

### MiR-615-3p reduces apoB secretion from human liver cells

**Fig. 2:**
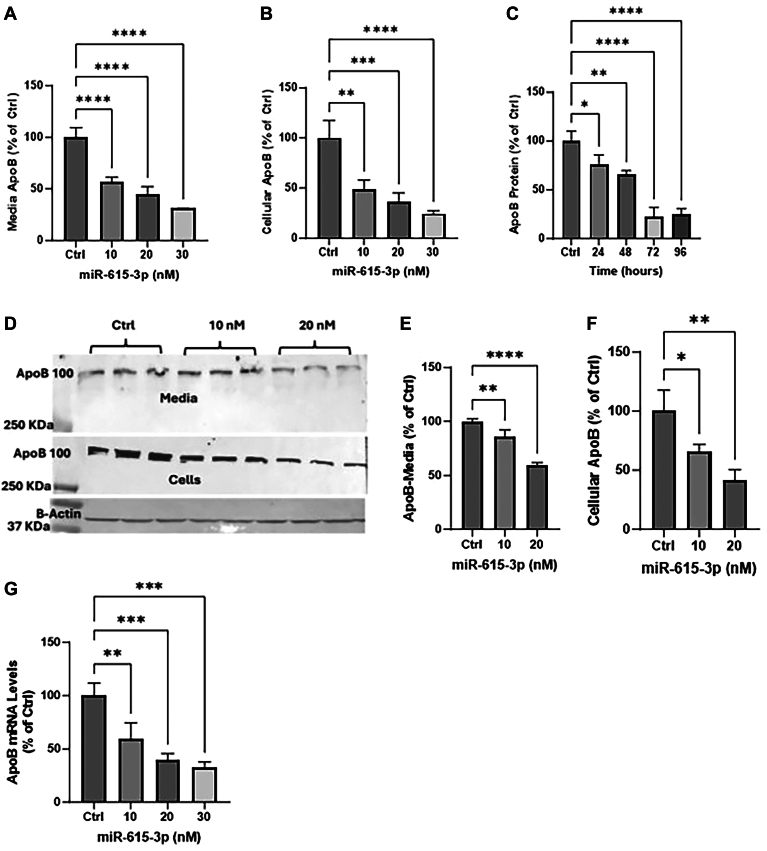
Downregulation of apoB by miR-615-3p. A, B: Huh-7 cells were reverse transfected with different indicated concentrations of miR-615-3p (n = 3). After 16 h, the media was changed. After 48 h, media was changed again, and overnight conditioned media was used to quantify apoB by ELISA (A). ApoB levels were normalized with cellular protein levels. ApoB levels in control cells were normalized to 100% and values in miR-615-3p treated cells were compared with those values. One-way ANOVA non-parametric or mixed test was performed to determine significance. After 70 h, cells were collected to quantify cellular apoB levels by ELISA (B). C: Huh-7 cells were transfected with 20 nM miR-615-3p. Media was collected at different times to quantify apoB protein levels by ELISA. D–F: Huh-7 cells were transfected with different indicated concentrations of miR-615-3p. ApoB in media (top) and cells (bottom) were visualized by Western blotting (D). For control, cellular β-actin levels were visualized. Bands were quantified using ImageJ, normalized and plotted (E, F). One-way ANOVA non-parametric or mixed test was performed to see the significance. G: after 48 h of transfection, Huh-7 cells were collected and used for RNA isolation using TRIzol. ApoB mRNA levels were quantified by qRT-PCR. ∗*P* < 0.05; ∗∗, *P* < 0.01, ∗∗∗*P* < 0.001; ∗∗∗∗*P* < 0.0001. One-way ANOVA non-parametric or mixed test. Data are representative of 3 independent experiments.

To determine whether miR-615-3p regulates apoB protein levels, we expressed it in human hepatoma Huh-7 cells. Increasing concentrations of miR-615-3p significantly decreased media (Fig. 2A) and cellular (Fig. 2B) apoB protein as measured by ELISA compared to control cells transfected with 30 nM Scr control miR. To find out the time course of reductions in media, apoB levels were quantified at different times after miR-615-3p expression. The highest reductions in apoB protein levels in media were seen after 72 h of transfection (Fig. 2C). Western blot analysis also revealed significant reductions in apoB100 in Huh-7 media and cells (Fig. 2D–F). In addition, miR-615-3p significantly reduced (∼78%) mRNA levels of apoB (Fig. 2G). These studies showed that overexpression of miR-615-3p reduces apoB mRNA and protein levels.

**Fig. 3:**
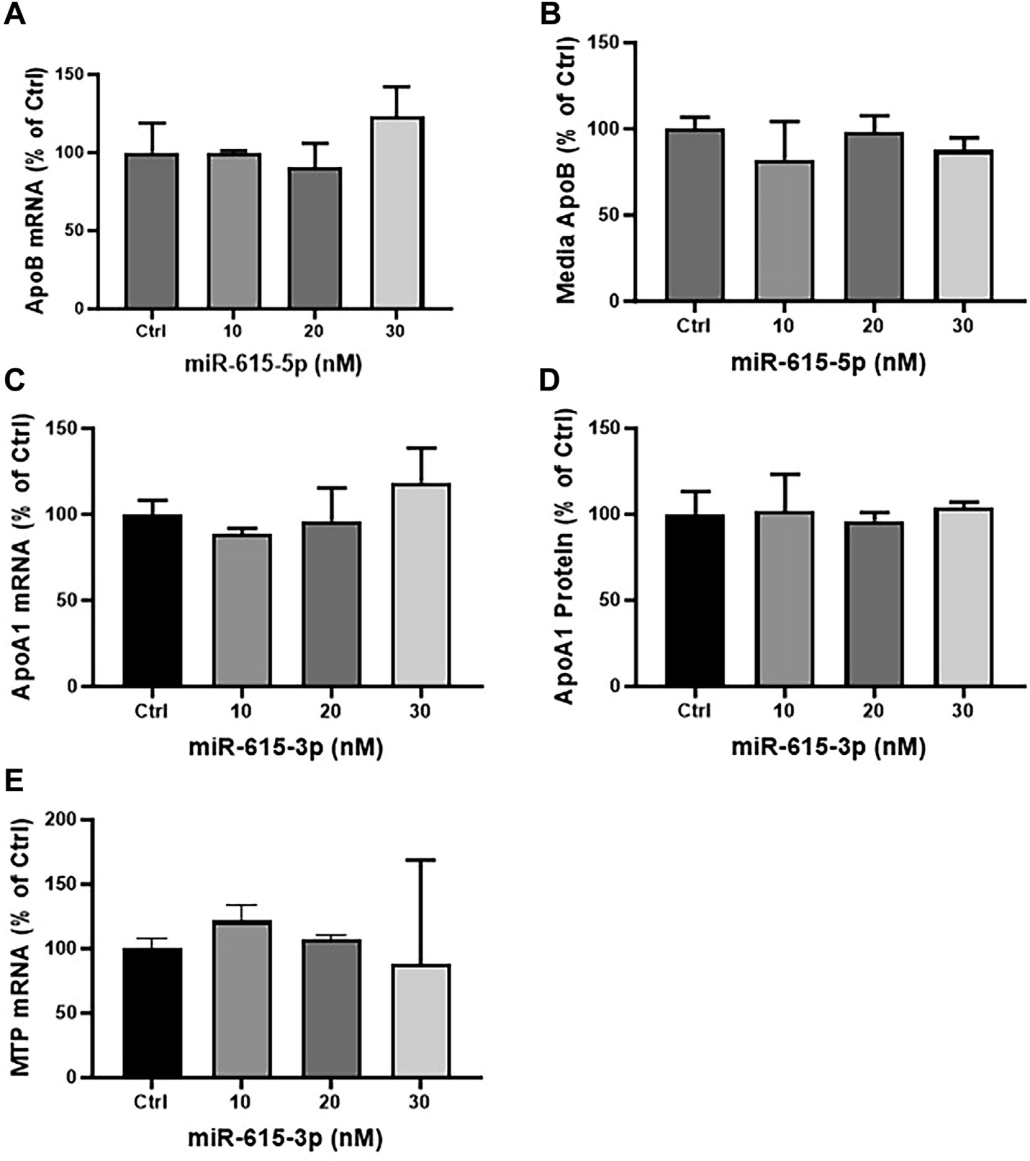
MiR-615-3p has no effect on apoA1 and MTP expression. A, B: Huh-7 cells were reverse transfected with miR-615-5p, the complementary strand of miR-651-3p. ApoB mRNA was quantified in cells (A) and protein in media (B). MiR-615-5p has no effect on apoB expression. C, D: cells were reverse transfected with different amounts of miR-615-3p. Cells were used to measure mRNA levels (C) and media was used to quantify apoA1 protein levels by ELISA (D). E: Huh-7 cells were reverse-transfected with different amounts of miR-615-3p. After 48 h, cells were used to quantify MTP (E) mRNA levels. MiR-615-3p has no significant effect on the expression of apoA1 and MTP. ∗*P* < 0.05; ∗∗, *P* < 0.01, ∗∗∗*P* < 0.001; ∗∗∗∗*P* < 0.0001. One-way ANOVA non-parametric test. Data are representative of 3 independent experiments.

Next, several experiments were performed to address the specificity of the regulation of apoB by miR-615-3p. First, we asked whether the complementary strand of miR-615-3p, ie miR-615-5p, also affects apoB mRNA levels. We found that miR-615-5p did not affect apoB mRNA and protein levels (Fig. 3A, B). Second, miR-615-3p did not affect the mRNA and protein levels of apoA1 (Fig. 3C, D). Third, we asked whether miR-615-3p affects the expression of other genes that could affect apoB secretion. Different concentrations of miR-615-3p did not affect mRNA levels of MTP (Fig. 3E). These studies suggest that miR-615-3p specifically regulates apoB expression in Huh-7 cells.

### Endogenous miR-615-3p regulates apoB secretion

**Fig. 4:**
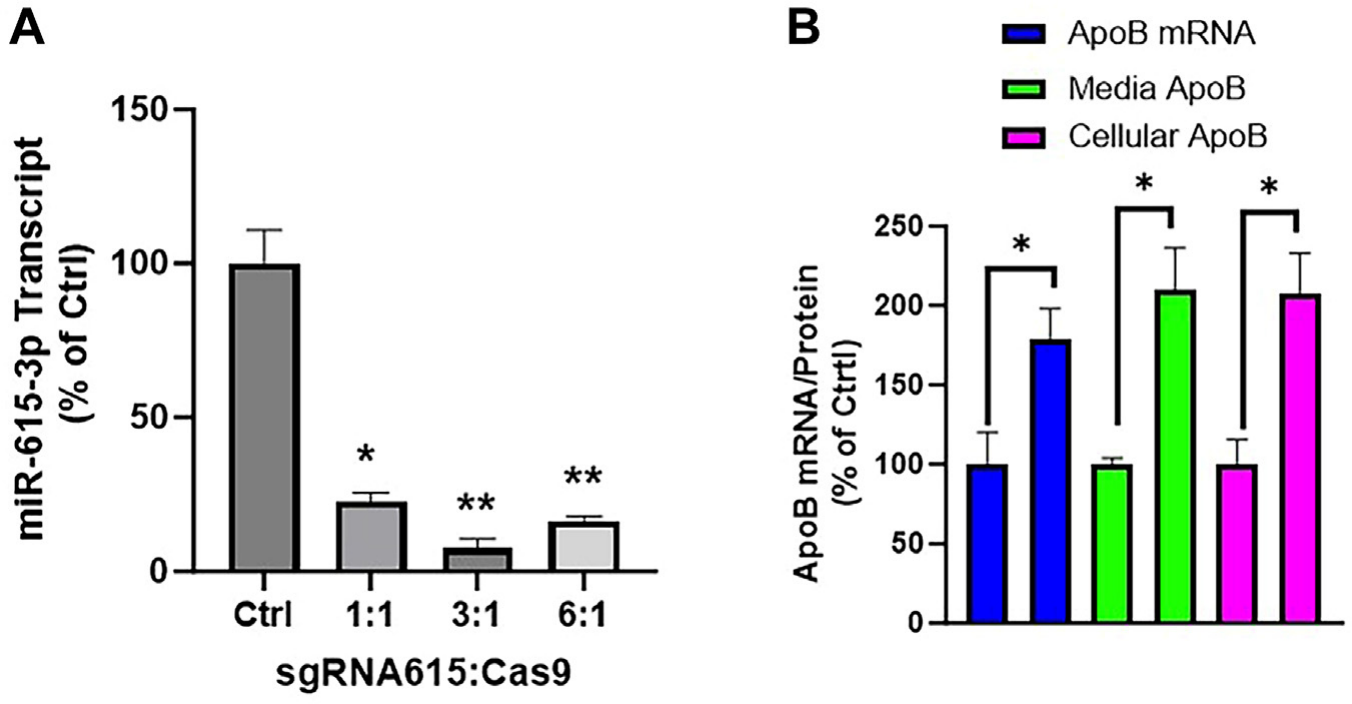
Endogenous miR-615-3p regulates apoB mRNA and protein levels. A: Huh-7 cells were electroporated (n = 3) with specific sgRNA-miR-615 or not (control, ctrl) along with Cas9 with different indicated ratios between gRNA and Cas9 protein. After 48 h, cellular miR-615-3p levels were quantified by Taqman. ∗*P* < 0.05; ∗∗, *P* < 0.01, One-way ANOVA compared with control. B: Huh-7 cells were electroporated (n = 3) with sgRNA-miR-615/Cas9 complexes at 3:1 ratio. Control cells were electroporated without sgRNA/Cas9 complexes. After 48 h, cells were used to measure apoB mRNA levels. After 70 h, cells and media were also used to measure apoB protein levels. ∗*P* < 0.05; ∗∗, *P* < 0.01, ∗∗∗*P* < 0.001; ∗∗∗∗*P* < 0.0001. Unpaired parametric Student's *t* test. Data are representative of 3 independent experiments.

Earlier studies showed that the expression of miR-615-3p reduces apoB expression. To determine whether endogenous miR-615-3p also reduces apoB expression, we knocked down endogenous miR-615-3p by electroporating a complex of Cas9 and a specific small guide RNA against *MIR615* (sgRNA-miR-615). We found that sgRNA-miR-615 significantly reduced (>90%) endogenous miR-615-3p (Fig. 4A) and increased apoB mRNA as well as media and cellular apoB protein levels (Fig. 4B). These studies indicated that endogenous miR-615-3p is a negative regulator of apoB expression and secretion.

### MiR-615-3p interacts with the 3′-UTR of apoB to induce post-transcriptional degradation

**Fig. 5:**
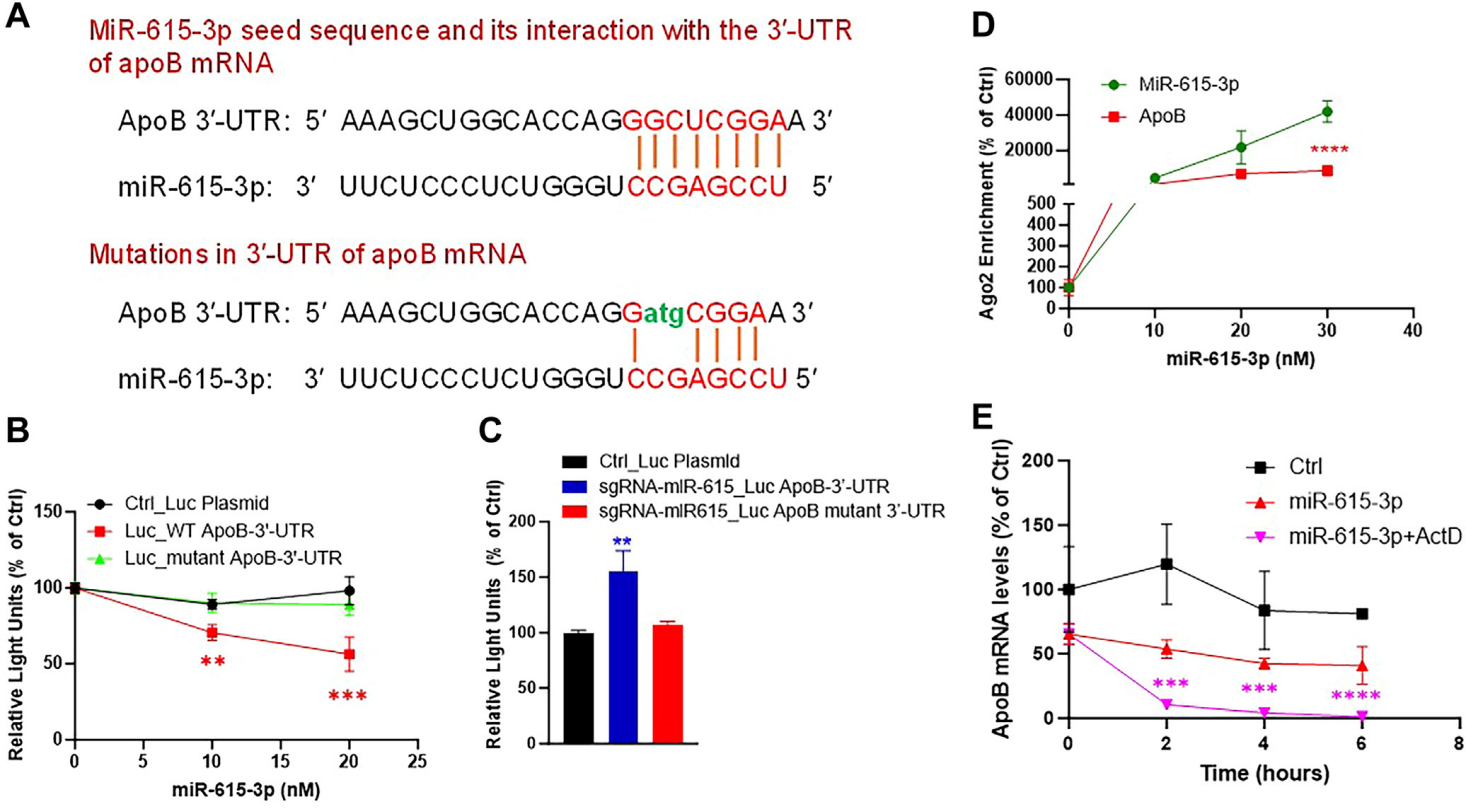
MiR-615-3p interacts with 3′-UTR of apoB mRNA and induces post-transcriptional degradation. A: a schematic representation showing base-pairing between apoB mRNA 3′-UTR and seed sequence of miR-615-3p involving 8 nucleotides (top). Mutations were made in the 3′-UTR of apoB mRNA in dual luciferase apoB plasmid as indicated in green to disrupt interactions between miR-615-3p seed sequence and human apoB mRNA 3′-UTR (bottom). B: Huh-7 cells were first forward transfected with different plasmids expressing luciferase with a control 3′-UTR (Ctrl_Luc), WT apoB 3′-UTR, or a mutant apoB 3′-UTR. After 24 h, cells were trypsinized and reverse transfected in 6-well plates with indicated concentrations of miR-615-3p complexed with Endofectin. After 8 h, media was changed. Conditioned media after 24 h was used to measure luciferase activity. C: Huh-7 cells were first forward transfected with plasmids expressing luciferase with control 3′-UTR, WT apoB 3′-UTR or mutant apoB 3′-UTR. After 24 h, cells were trypsinized and electroporated with or without sgRNA-miR-615 in triplicate. After 24 h, media was used to measure luciferase activity. Data from cells subjected to electroporation without sgRNA were used as controls. D: Huh-7 cells were transfected with different amounts of miR-615-3p (n = 3). After 48 h, Ago2 complexes were immunoprecipitated and used to quantify apoB mRNA and miR-615-3p. Control cells transfected with no miR were normalized to 100% to compare cellular ApoB mRNA and miR-615-3p levels. ∗∗∗∗*P* < 0.001, two-way ANOVA. E: Huh-7 cells were transfected in triplicate with 20 nM control miR or miR-615-3p. After 24 h, miR-615-3p transfected cells were washed and treated with 10 μg/ml of actinomycin D (miR-615-3p+ActD) or not (miR-615-3p). At different times, cells were collected to quantify apoB mRNA levels. ∗∗∗*P* < 0.001; ∗∗∗∗*P* < 0.0001. One-way ANOVA non-parametric test. Representative of 3 different experiments.

MiRs generally interact with the 3′-UTR of target mRNAs mainly through their seed sequences (17, 31, 32). We found a potential 8-mer miR-615-3p seed complementary sequence in human apoB mRNA 3′-UTR (Fig. 5A, top) suggesting strong interactions between miR-615-3p and apoB mRNA. To address the hypothesis that interactions between miR-615-3p and 3′-UTR of apoB mRNA are critical for the regulation of apoB by miR-615-3p, we purchased a plasmid for the expression of luciferase with 3′-UTR of apoB mRNA. Next, three nucleotides in the apoB 3′-UTR of this plasmid were mutated (Fig. 5A, bottom). First, to study the effects of miR-615-3p on luciferase expression, Huh-7 cells were transfected with plasmids for the expression of a control luciferase with unrelated 3′-UTR sequence, luciferase with wild-type apoB 3′-UTR or luciferase with mutant apoB 3′-UTR (Fig. 5B). These cells were then treated with different amounts of miR-615-3p. MiR-615-3p had no effect on the luciferase expression when plasmids contained a control unrelated 3′-UTR or a mutant apoB 3′-UTR. However, the expression of luciferase was significantly reduced by different concentrations of miR-615-3p when the plasmid contained wild-type apoB 3′-UTR (Fig. 5B). These studies showed that interactions between complementary sequences in the 3′-UTR of apoB mRNA with the seed sequence of miR-615-3p are important for apoB regulation.

Next, we studied the role of endogenous miR-615-3p on the expression of luciferase with wild-type and mutant apoB 3′-UTR sequence (Fig. 5C). Expression of luciferase with wild-type apoB 3′-UTR was significantly increased after miR-615-3p knockdown (Fig. 5C). However, miR-615-3p knockdown had no effect on the expression of luciferase with mutant apoB 3′-UTR compared to control luciferase. These studies demonstrated that seed complementary sequences in the apoB mRNA facilitate miR-615-3p-induced apoB repression.

It is known that miRs and mRNAs interact with each other in Ago2 protein containing RISC complexes (16, 31, 33, 34, 35, 36, 37, 38). Huh-7 cells were transfected with different concentrations of miR-615-3p and used to immunoprecipitate Ago2 complexes. Quantification of apoB and miR-615-3p in the Ago2 complexes revealed that transfections of increasing amounts of miR-615-3p into Huh-7 cells increased the amounts of both apoB mRNA and miR-615-3p in these complexes (Fig. 5D). These investigations suggest that the levels of apoB mRNA and miR-615-3p in RISC complexes rise as the concentrations of miR-615-3p increase.

Interactions between miRs and mRNAs usually lead to posttranscriptional degradation of target mRNAs (31, 33, 37). Therefore, we addressed whether miR-615-3p increases the degradation of apoB mRNA (Fig. 5E). Cells transfected with miR-615-3p were treated with or without actinomycin D to inhibit transcription, and apoB mRNA levels were quantified at different times to follow post-transcriptional degradation. In control cells that were not treated with miR-615-3p or actinomycin D, apoB mRNA levels did not decrease much during the study indicating for long half-life (Fig. 5E). ApoB mRNA levels were less in miR-615-3p treated cells than in control cells consistent with the observations that miR-615-3p reduces apoB mRNA. ApoB mRNA degradation was faster in cells transfected with miR-615-3p compared to control cells indicating that miR enhances apoB degradation. This degradation was significantly enhanced in cells also treated with actinomycin D suggesting that post-transcriptional degradation of apoB mRNA is enhanced in miR-615-3p expressing cells.

### MiR-615-3p increases cellular triglyceride levels

**Fig. 6:**
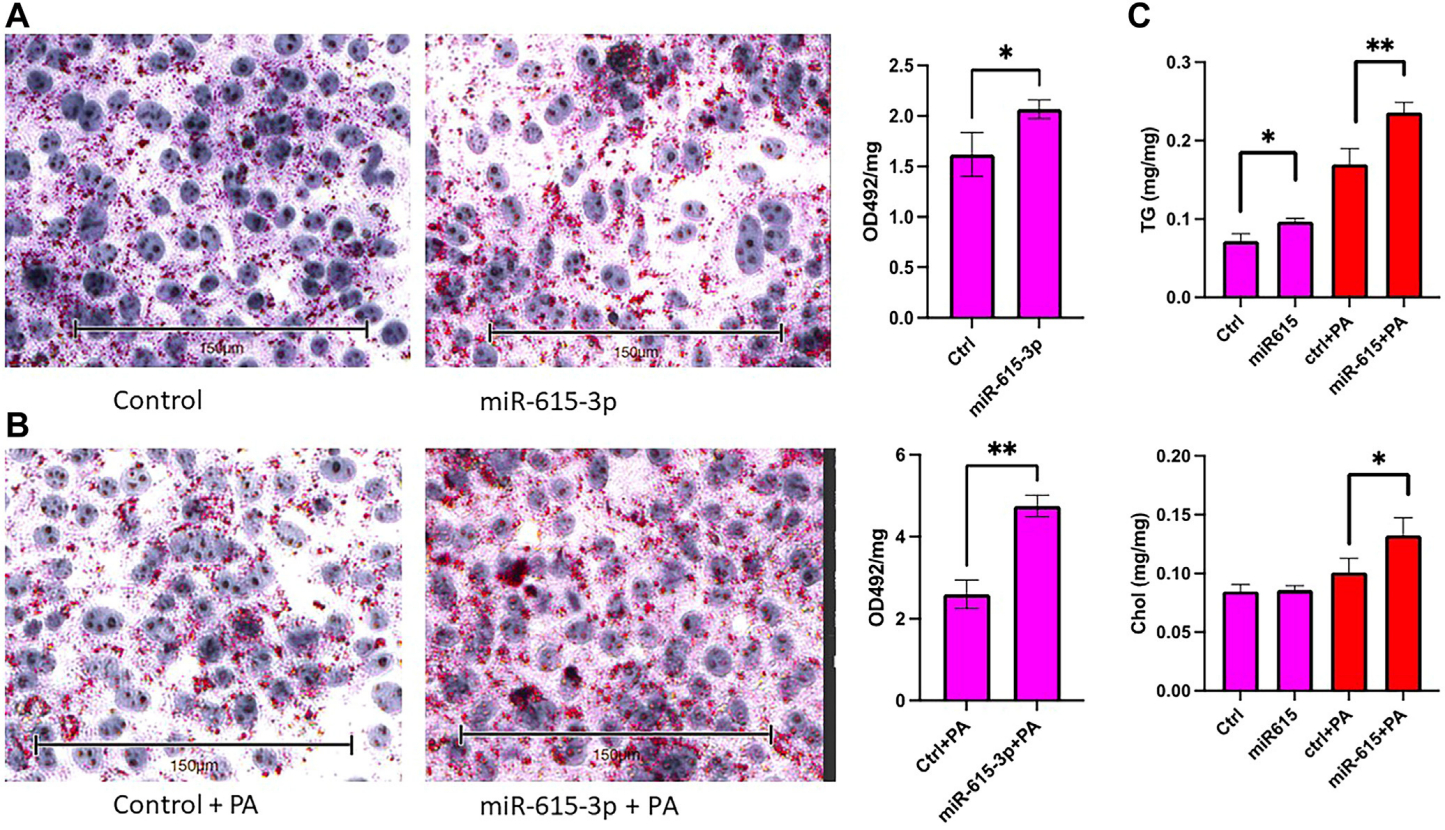
MiR-615-3p increases cellular neutral lipids. A–C: Huh-7 cells were transfected in triplicate with control miR or miR-615-3p [25 nM]. After 72 h, cells received palmitic acid (PA, 0.5 mM) complexed with BSA or BSA alone. After 8 h incubation, cells were stained with Oil Red O followed by hematoxylin staining and photographed (A, B). Subsequently, the dye was extracted using isopropanol and quantified. Cells were collected in 0.1 N NaOH and quantified. Absorbance values normalized to cellular protein levels. ∗*P* < 0.05. Data are representative of 2 independent experiments. C: Huh-7 cells were transfected in triplicate with control miR or miR-615-3p. After 72 h, cells were treated with or without PA (0.5 mM). After 8 h, cells were washed and lipids were extracted using isopropanol followed by protein extraction with 0.1 N NaOH. TG, triglyceride; chol, cholesterol. ∗ and ∗∗, *P* < 0.05 and 0.01, respectively.

The studies described so far show that miR-615-3p decreases apoB expression and secretion. Since reductions in apoB secretion have been shown to increase cellular triglyceride levels in Huh-7 cells (28), we asked whether miR-615-3p expression affects cellular triglyceride levels. Since miR-615-3p has been implicated in saturated fatty acid-induced lipoapoptosis of liver cells (39), we also studied the effects of palmitic acid in control miR and miR-615-3p transfected cells. Oil Red O staining showed that expression of miR-615-3p increases neutral lipid levels (Fig. 6A). Similarly, cells transfected with miR-615-3p and treated with palmitic acid showed more lipid staining (Fig. 6B). Furthermore, we measured cellular mass of triglyceride and cholesterol and found that miR-615-3p significantly increased cellular triglyceride but had no effect on cellular cholesterol content (Fig. 6C). PA treatment increased cellular triglyceride and cholesterol levels in control and miR-615-3p transfected cells. These studies suggest that overexpression of miR-615-3p increases cellular TG levels and these levels are increased further after PA supplementation.

### ApoB expression is further reduced by palmitic acid in miR-615-3p-expressing cells

**Fig. 7:**
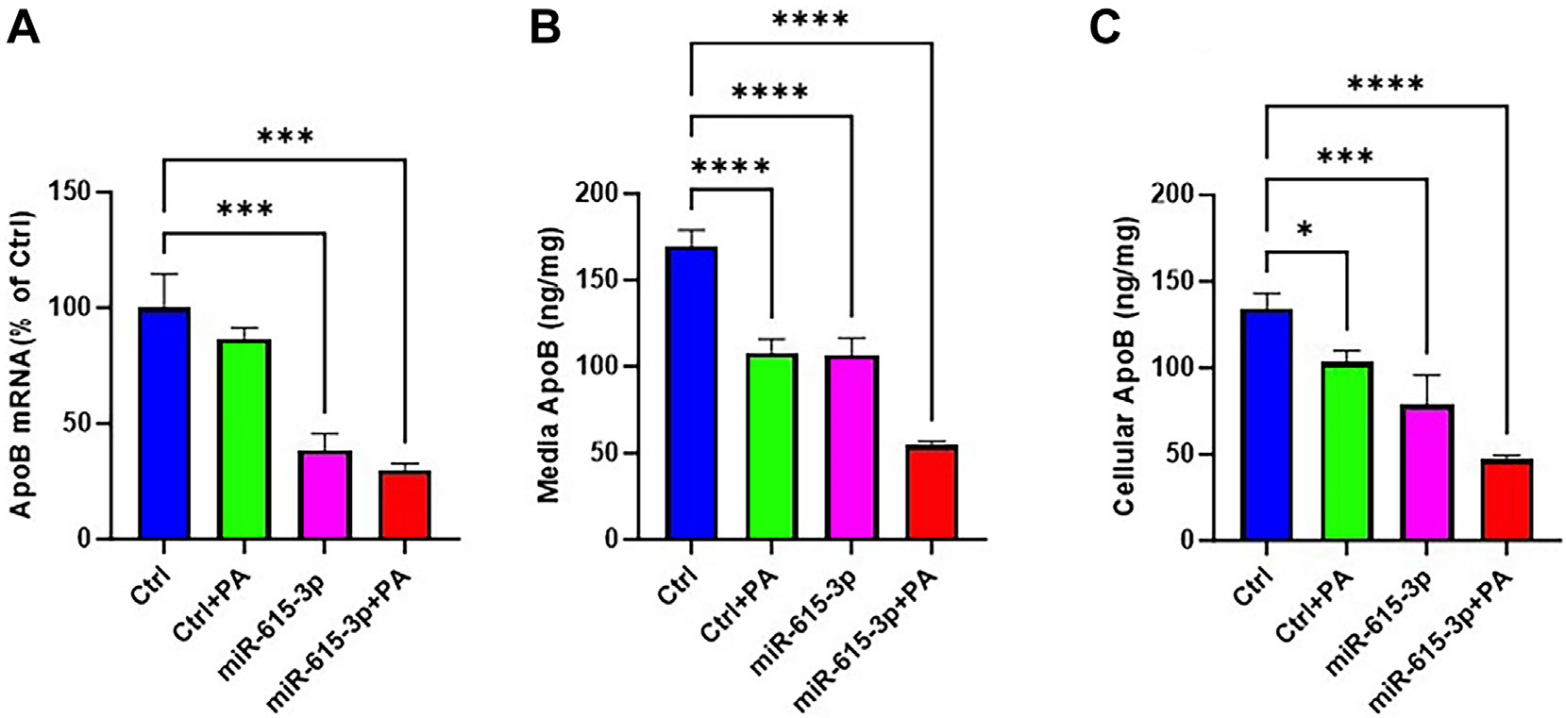
Palmitic acid reduces apoB expression in miR-615-3p expressing cells. A: Huh-7 cells were transfected with control miR or miR-615-3p [25 nM]. After 40 h, cells were treated with palmitic acid [0.5 mM] for 8 h. Cells were then used to measure cellular apoB mRNA levels. ∗, ∗∗, and ∗∗∗∗ represent *P* < 0.05, 0.01, and 0.001, respectively. Student *t* test. Data are representative of 2 independent experiments. B, C: Huh-7 cells were transfected with control miR or miR-615-3p (25 mM). After 40 h, media was changed and supplemented with palmitic acid (0.5 mM). After 8 h, media (B) and cellular (C) apoB levels were quantified by ELISA. Cellular and media apoB were significantly reduced in cells transfected with miR-615-3p and then treated with PA. ∗, ∗∗, and ∗∗∗∗ represent *P* < 0.05, 0.01, and 0.001, respectively. Student *t* test.

Since cellular triglyceride levels increased in the presence of PA, we asked whether PA affects apoB expression in miR-615-3p overexpressing cells. We observed that PA treatment did not affect apoB mRNA levels in control Huh-7 cells (Fig. 7A). As expected, miR-615-3p significantly reduced apoB mRNA levels. A combination of miR-615-3p and PA appeared to decrease apoB mRNA more than miR-615-3p alone. Next, we studied the effect of miR-615-3p and PA on apoB protein levels in media and cells. In contrast to mRNA levels, PA alone significantly reduced media and cellular apoB levels (Fig. 7B, C). As shown before, miR-615-3p reduced cellular and secreted apoB (Fig. 7B, C). More importantly, PA and miR-615-3p appear to reduce additively media and cellular apoB levels. These studies indicate that PA and miR-615-3p individually reduce media and cellular apoB levels and when present together might act additively to further reduce apoB protein levels suggesting that PA and miR-615-3p regulate apoB synthesis and secretion via independent mechanisms.

### MiR-615-3p does not induce endoplasmic reticulum stress in Huh-7 cells

**Fig. 8:**
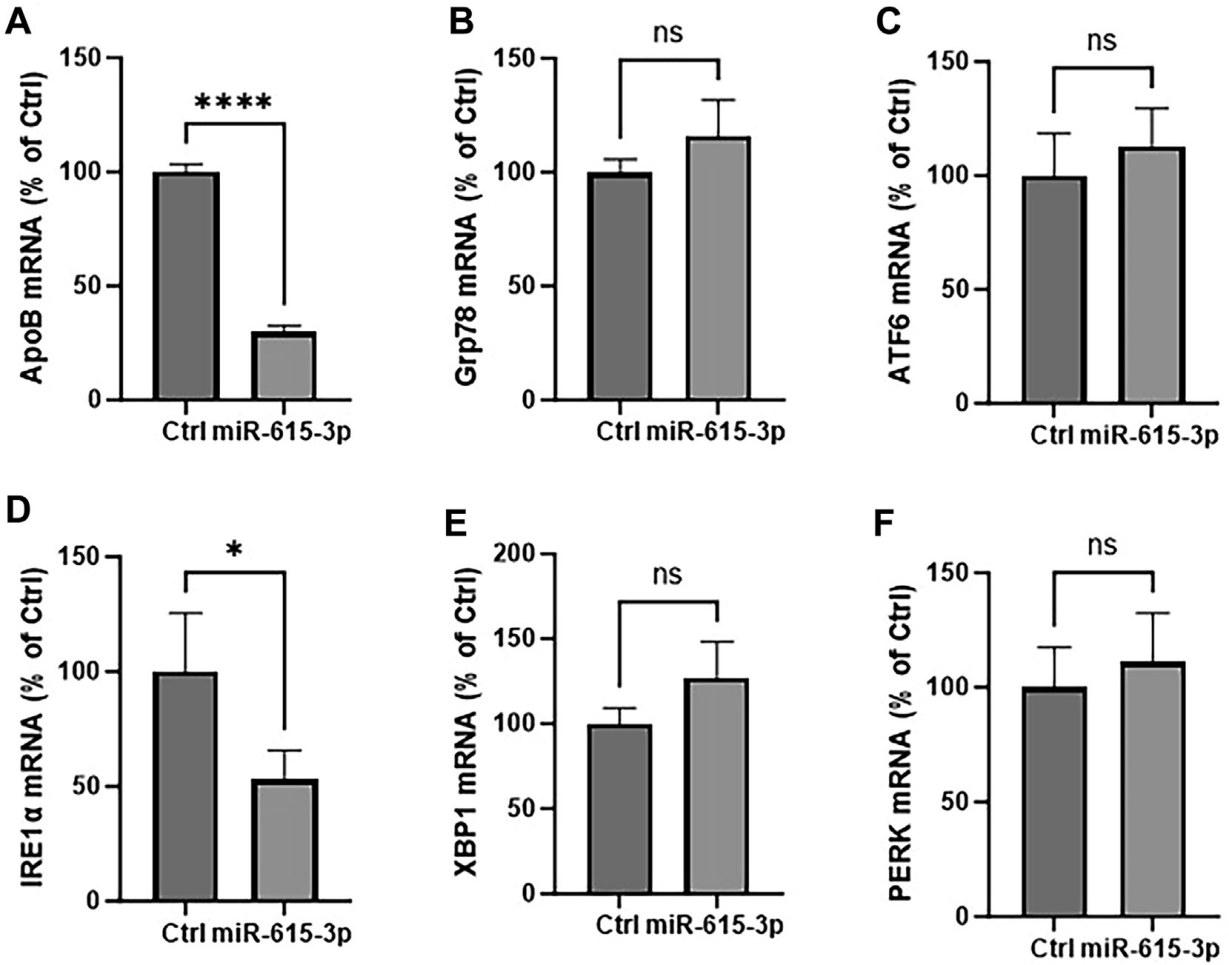
MiR-615-3p does not induce ER stress. Huh-7 cells were transfected in triplicate with control miR or miR-615-3p (25 mM). After 40 h, media was changed and supplemented with palmitic acid (0.5 mM). After 8 h, total RNA was isolated and mRNA levels of different ER stress marker genes (A–F) were quantified. ∗ and ∗∗∗∗ represent *P* < 0.05 and 0.001, respectively. Student *t* test.

Endoplasmic reticulum (ER) stress is associated with reduced TG secretion from liver cells ultimately resulting in lipoapoptosis. Since miR-615-3p reduced apoB secretion and increased cellular triglyceride, we asked whether elevated miR-615-3p expression can lead to ER stress. Quantifications of major ER stress markers at the mRNA level did not show any significant changes except for a significant reduction in Ire-1α mRNA (Fig. 8). Therefore, these data suggest that miR-615-3p does not induce ER stress under these conditions.

## Discussion

The *MIR615* gene located within an intron of *HOXC5* gene is conserved only in eutherian mammals suggesting its role in the evolution and development of placental mammals. Based on gene sequence analysis, it can be inferred that the *MIR615* gene can express either in conjunction with *HOXC5* or independently of *HOXC5* expression due to the presence of an intronic promoter. In addition, the expression can be affected by hypermethylation of its intragenic promoter (40). The pre-mir-615 is 96 nucleotide-long RNA that could potentially form stem-loop structures and give rise to mature miR-615-3p and miR-615-5p microRNAs. Apart from transcriptional and epigenetic regulation, several competing endogenous RNAs, such as IncRNAs and circ-RNAs, have been identified that could modulate the activity of miR-615-3p. Thus, transcription, epigenetic regulation, and inhibition by binding to competing endogenous RNAs can modulate the expression and activity of miR-615-3p (40).

In cellular studies, miR-615-3p has been shown to regulate osteogenic and chondrogenic differentiation, tumor progression and regression, phagocytic capacity of splenic macrophages, as well as hypoglycemic and hypoxic stress-induced umbilical vein endothelial cell apoptosis and angiogenesis (40). Few targets of miR-615-3p potentially mediating its functions have been identified. For example, miR-615-3p has been shown to downregulate Sox9 in chondrocytes and inhibit chondrogenic differentiation (41). A systematic review and meta-analysis identified higher miR-615-3p levels in body fluids as selective biomarkers and therapeutic targets for Alzheimer’s, but not for Parkinson’s, disease (42). Peripheral blood from acute respiratory syndrome has high levels of miR-615-3p and several inflammatory cytokines. Therefore, it has been suggested that miR-615-3p can help the progression of the disease (43). Furthermore, miR-615-3p has been implicated in saturated fatty acid-induced lipoapoptosis of liver cells (39). Palmitate reduces the expression of miR-615-3p and increases lipoapoptosis in liver cells. However, very little is known about the role of miR-615-3p in the regulation of plasma lipids, lipoproteins, and its association with metabolic diseases.

This study describes a novel function of miR-615-3p in the regulation of apoB in human liver cells. We show that overexpression of miR-615-3p reduces apoB mRNA, as well as intracellular and secreted apoB protein levels. Mechanistic studies suggest that the seed sequence of miR-615-3p interacts with the 3′-UTR of apoB mRNA in RISC complexes and enhances post-transcriptional mRNA degradation leading to reduced cellular and secreted apoB. MiR-615-3p increases cellular triglyceride levels and these accumulations are further enhanced after PA supplementation. However, these changes were not associated with ER stress.

Although miR-615-3p is broadly conserved microRNA among mammals, bioinformatics analysis revealed that miR-615-3p only interacts with human apoB mRNA 3′-UTR. This suggests that recognition of apoB mRNA by miR-615-3p might a be recent evolutionary acquisition and that the role of miR-615-3p in the control of metabolic pathways is expanding in humans. More in-depth evolutionary studies across different species may uncover a pattern about the acquisition of new abilities of this miR to regulate different pathways.

ApoB lipoproteins are synthesized and secreted by the liver and the intestine to deliver endogenous and dietary to peripheral tissues. Excess accumulation of modified apoB-lipoprotein in the plasma, and their uptake by macrophages, contributes to ASCVD. Thus, targeting the liver ApoB could have beneficial effects on reducing atherosclerosis. Successful attempts have been made to reduce apoB secretion using anti-sense approaches. It is likely that miR-615-3p can also be used to reduce plasma apoB-containing lipoproteins; however, these approaches are likely to enhance hepatic steatosis. Therefore, apoB targeting efforts must be used in combination with other reagents that could reduce lipid synthesis, increase fatty acid oxidation, or both.

ApoB synthesis and secretion are mainly regulated at co-translational and post-transcriptional levels involving ER-associated degradation as well as post-ER degradation mechanisms (2, 44, 45, 46). Because earlier studies showed that apoB mRNA levels do not change under different conditions (47), it has been assumed that transcriptional and post-transcriptional mechanisms may not play a role in the regulation of apoB synthesis and secretion. However, recent studies have started to highlight the roles of different RNA-binding proteins in the synthesis of apoB (13). Our group has been focusing on post-transcriptional mechanisms that control the assembly and secretion of apoB-containing lipoproteins. We identified miR-548p that interacts with and degrades apoB mRNA (14). We have also shown that miR-541-3p indirectly reduces apoB mRNA by diminishing the expression of an apoB transcriptional enhancer, Znf101 (26). Here, we reveal miR-615-3p as yet another major post-transcriptional regulator of apoB mRNA degradation affecting apoB secretion. Therefore, it is likely that more microRNAs control apoB than previously anticipated, and their discovery may identify novel mechanisms involved in post-transcriptional control of apoB secretion. In short, emerging evidence indicates that transcriptional and post-transcriptional mechanisms also regulate the assembly and secretion of apoB-containing lipoproteins. These novel mechanisms and genes involved in transcription and post-transcriptional regulation of apoB synthesis may provide new opportunities for novel therapies to lower plasma LDL levels.

It has been shown that PA reduces miR-615-3p and this might be a mechanism to increase lipoapoptosis. However, our studies indicate that PA additively increases cellular lipid accumulation, therefore, over-expression of miR-615-3p might not be a suitable approach to reduce lipoapoptosis. It is likely that PA-mediated lipoapoptosis is not related to reduced levels of miR-615-3p. However, more focused and in-depth analyses are needed to prove or disprove this hypothesis, as our experiments were not designed to address them directly.

We had anticipated that cellular lipid retention and reduced apoB secretion would increase ER stress. However, we did not observe any significant increase in mRNA levels of select markers of ER stress. It remains to be determined why decreases in apoB secretion and increases in cellular lipids in miR-615-3p expressing cells do not induce ER stress. It is possible that miR-615-3p might be involved in suppressing the expression of ER stress genes.

Besides the liver, another tissue that expresses apoB is the intestine. We have not studied the effect of miR-615-3p in the intestine. Future studies may unravel whether miR-615-3p can also reduce apoB secretion in enterocytes or it is a tissue-specific effect. Based on the mechanisms of action of miR via its interaction with apoB mRNA, we speculate that miR-615-3p would also reduce apoB secretion in enterocytes.

In short, these studies identify miR-615-3p as a new regulator of apoB mRNA and protein levels. It interacts with 3′-UTR of apoB mRNA in the RISC complex to enhance post-transcriptional degradation. This leads to lower cellular levels and reduced secretion of apoB. In addition, miR-615-3p increased cellular triglyceride levels both in the presence and absence of PA. This miR can be used to lower plasma lipoproteins in conjunction with other reagents that can reduce hepatic steatosis Identification of this miR suggests that additional miRs exist that regulate apoB-containing lipoprotein secretion. Identification of additional novel miRs may uncover novel mechanisms that regulate lipoprotein assembly and secretion.

## Data availability

All the data are in the paper.

## Conflicts of interest

The authors declare that they have no conflicts of interest with the contents of this article.
